# Prevalence of *Plasmodium falciparum* and Non-*falciparum* Infections by Photo-Induced Electron Transfer–PCR in a Longitudinal Cohort of Individuals Enrolled in a Mass Drug Administration Trial in Southern Province, Zambia

**DOI:** 10.4269/ajtmh.19-0668

**Published:** 2020-07-02

**Authors:** Sandra Chishimba, Mulenga Mwenda, Brenda Mambwe, Conceptor Mulube, Victor Chalwe, Hawela Moonga, Busiku Hamainza, Elizabeth Chizema-Kawesha, Richard W. Steketee, Gonzalo Domingo, Maya Fraser, Maria Kahn, Sampa Pal, Kafula Silumbe, Ruben O. Conner, Adam Bennett, Travis R. Porter, Thomas P. Eisele, John M. Miller, Daniel J. Bridges

**Affiliations:** 1 PATH Malaria Control and Elimination Partnership in Africa (MACEPA), Lusaka, Zambia;; 2 Zambia Ministry of Health Provincial Medical Office, Mansa, Zambia;; 3 National Malaria Elimination Centre, Zambia Ministry of Health, Lusaka, Zambia;; 4 PATH Malaria Control and Elimination Partnership in Africa (MACEPA), Seattle, Washington;; 5 Malaria Elimination Initiative, Global Health Group, University of California San Francisco, San Francisco, California;; 6 Department of Tropical Medicine, Center for Applied Malaria Research and Evaluation, Tulane University School of Public Health and Tropical Medicine, New Orleans, Louisiana

## Abstract

Malaria burden in Zambia has significantly declined over the last decade because of improved coverage of several key malaria interventions (e.g., vector control, case management, bed net distributions, and enhanced surveillance/responses). Campaign-based mass drug administration (MDA) and focal MDA (fMDA) were assessed in a trial in Southern Province, Zambia, to identify its utility in elimination efforts. As part of the study, a longitudinal cohort was visited and tested (by PCR targeting the 18s rRNA and a *Plasmodium falciparum*–specific rapid diagnostic test [RDT] from SD Bioline) every month for the trial duration (18 months). Overall, there was high concordance (> 97%) between the PCR and RDT results, using the PCR as the gold standard. The RDTs had high specificity and negative predictive values (98.5% and 98.6%, respectively) but low sensitivity (53.0%) and a low positive predictive value (53.8%). There was evidence for persistent antigenemia affecting the low specificity of the RDT, while false-negative RDTs were associated with a lower parasite density than true positive RDTs. *Plasmodium falciparum* was the dominant species identified, with 98.3% of all positive samples containing *P. falciparum*. Of these, 97.5% were mono-infections and 0.8% coinfections with one other species. *Plasmodium malariae* was found in 1.4% of all positive samples (50% mono-infections and 50% coinfections with *P. falciparum*), whereas *Plasmodium ovale* was found in 1.1% of all positive samples (90% mono-infections and 10% coinfections with *P. falciparum*). Although MDA/fMDA appeared to reduce *P. malariae* prevalence, *P. ovale* prevalence appeared unchanged.

## INTRODUCTION

Buoyed by dramatic reductions in malaria morbidity and mortality, Zambia has recently adopted a strategy aimed at achieving national elimination by 2021.^1^ The strategy includes the use of mass drug administration (MDA) to accelerate to zero transmission. Although historically MDA was found to be a useful malaria control tool, having a substantial short-term impact on parasite prevalence, it fell out of favor predominantly because of concerns around drug resistance and, once it was withdrawn, resurgence. More recently, the utility of MDA has been revisited, although its optimal application and cost-benefit remain under debate.^2^ Nevertheless, MDA has been shown to be effective in Zambia under research conditions, especially in lower transmission settings,^3^ and is now being deployed at a large scale as part of national elimination efforts.

As transmission falls and elimination becomes the goal, it is necessary to ensure that all infections that maintain *R*_0_ above one, whether symptomatic or not, are cleared. Attacking the sub-patent asymptomatic reservoir may help achieve elimination more rapidly.^4^ A key challenge in treating all infections is identifying them. Compared with the most sensitive diagnostic tests such as loop-mediated isothermal amplification (LAMP)^5^ or PCR,^6^ current routine diagnostics such as microscopy and malaria rapid diagnostic tests (RDTs) have a higher limit of detection (LOD). Below this limit, infections may be missed, contributing significantly to transmission.^7^ Presumptive treatment (i.e., providing an antimalarial in the absence of test confirmation) mitigates for imperfect diagnostics and can provide community-level protection from reinfection for the duration of the drug’s half-life. To quantify the level of missed infections by routine diagnostic tests, a more sensitive tool such as PCR or LAMP is required. There are a large number of PCR assays for detection of malaria infections, each with a different LOD, sensitivity, specificity, cost, reproducibility, etc. The multiplex photo-induced electron transfer (PET)-PCR assay was used in this study, as it was developed specifically for large-scale surveillance in resource-limited settings.^8,9^

This study aimed at assessing the prevalence of all malaria infections as well as the RDT and PCR concordance for *P. falciparum* in a Zambian population experiencing low to very low transmission, that is, < 200 cases per 1,000 population, in the context of a community randomized controlled trial assessing the impact of MDA with dihydroartemisinin–piperaquine.

## MATERIALS AND METHODS

### Study design and sample collection.

As published previously,^10^ 30 high-transmission and 30 low-transmission health facility catchment areas (HFCAs) were randomly assigned to one of three arms of the study: MDA, focal MDA (fMDA), and control (*n* = 10 HFCAs in each). Within each of the 60 total HFCAs, roughly 40 individuals older than 3 months were enrolled in a nested longitudinal cohort and surveyed monthly by community health workers for the duration of the study (18 months).^11^ At each visit, the following were ascertained: finger-prick blood samples for Giemsa microscopy (first 6 months only), an RDT (SD Bioline Malaria Ag *P.f*, Standard Diagnostics, Gyeonggi-do, Republic of Korea), and dried blood spot (DBS). Individuals testing positive were treated with the national standard of care. Dried blood spots were collected on Whatman 903 protein saver cards (first 6 months) or Whatman no. 3 filter paper (remainder of study) (Whatman^®^, Maidstone, United Kingdom), dried during collection, packed in standard plastic “pill” bags with desiccants, and stored (< 1 month) at room temperature before being transported to the laboratory at the National Malaria Control Centre in Lusaka, Zambia, for storage at −20°C.

### DNA extraction.

DNA was extracted from a single 6-mm punch (equivalent to roughly 13 µL of blood) from each DBS using the QIAamp DNA mini kit (QIAGEN, Hilden, Germany) and eluted in 100 µL of buffer. Punches were cleaned between samples by dipping in 70% ethanol and flaming. Rapid diagnostic test–negative samples with two or more DBSs were extracted in pools of 10 or five (depending on RDT positivity of the area), whereas RDT-positive/PCR-pool–positives or single-spot DBSs were extracted individually. Extracted DNA was stored at 4°C for immediate analysis or at −20°C for longer term storage.

### PCR detection.

Extracted parasite DNA was detected using PET-PCR,^12^ on a LightCycler 480 real-time PCR machine (Roche, Basel, Switzerland). In brief, all samples were tested in duplicate (5 µL of template, equivalent to 0.7 µL of whole blood) in a duplex reaction with *Plasmodium* spp. and *Plasmodium falciparum* primers labeled with FAM and HEX fluorophores, respectively. Samples with duplicate crossing point values of < 40 were recorded as positive. All samples positive by PET-PCR for *P. falciparum* or for other *Plasmodium* spp. at the genus level were then tested for the presence of other species, that is, *Plasmodium ovale*, *Plasmodium malariae*, and *Plasmodium vivax*, as described previously.^12^ A limiting dilution series of 3D7 *P. falciparum* genomic DNA (MRA-151G, ATCC, Manassas, VA), obtained through BEI Resources, National Institute of Allergy and Infectious Diseases (NIAID), National Institutes of Health (NIH), contributed by David Walliker of a known parasitemia was assayed three times in duplicate by PET-PCR. The standard curves generated from this series established a comparable LOD, as previously published.^12^ For clarity, any reference to PCR refers to PET-PCR, as no other PCR assay was used.

### Data analysis.

Data were aggregated using an Alteryx (Irvine, CA) workflow and visualized in Tableau (Seattle, WA) software. Standard curves relating crossing point values to parasitemia were fit using a linear regression model in R (Vienna, Austria). Odds ratios of outcomes in the current month based on the previous month were calculated in *R* using logistic regression.

## RESULTS

### *Plasmodium falciparum* detection.

DNA was extracted from 32,848 DBS samples and assessed for the presence of *P. falciparum* parasites by PCR. Of these, 31,492 had a valid RDT result, and of these, 10,696 had a valid microscopy reading. Using PCR as the gold standard, the sensitivity, specificity, and positive and negative predictive values of the RDT were assessed (Table 1). Whereas specificity (98.5%) and negative predictive values (NPVs, 98.6%) were as per the manufacturer’s expectations (roughly 98.5%), the sensitivity (54.2%) and positive predictive values (PPVs, 53%) of the RDT were significantly lower than the manufacturer’s reported performance of 93.8% sensitivity with 1–50 parasites/µL and 100% sensitivity at > 51 parasites/µL. A similar analysis of microscopy performance showed markedly lower sensitivity at 28.8%, albeit with roughly comparable specificity at 99.2% (Table 2).

**Table 1:** Comparison of RDT detection of *Plasmodium falciparum* infections against PCR as the gold standard for all mass drug administration cohort samples with results for both tests

	PCR+	PCR−	
RDT+	503	446	53.0% PPV
RDT−	425	30,117	98.6% NPV
	54.2% sensitivity	98.5% specificity	–

PPV = positive predictive value; NPV = negative predictive value; RDT = rapid diagnostic test.

**Table 2:** Comparison of microscopy detection of *Plasmodium falciparum* infections against PCR as the gold standard for all mass drug administration cohort samples with results for both tests

	PCR+	PCR−	
Microscopy+	130	32	80.2% PPV
Microscopy−	322	10,212	96.9% NPV
	28.8% sensitivity	99.7% specificity	–

PPV = positive predictive palue; NPV = negative predictive value.

**Figure 1.**
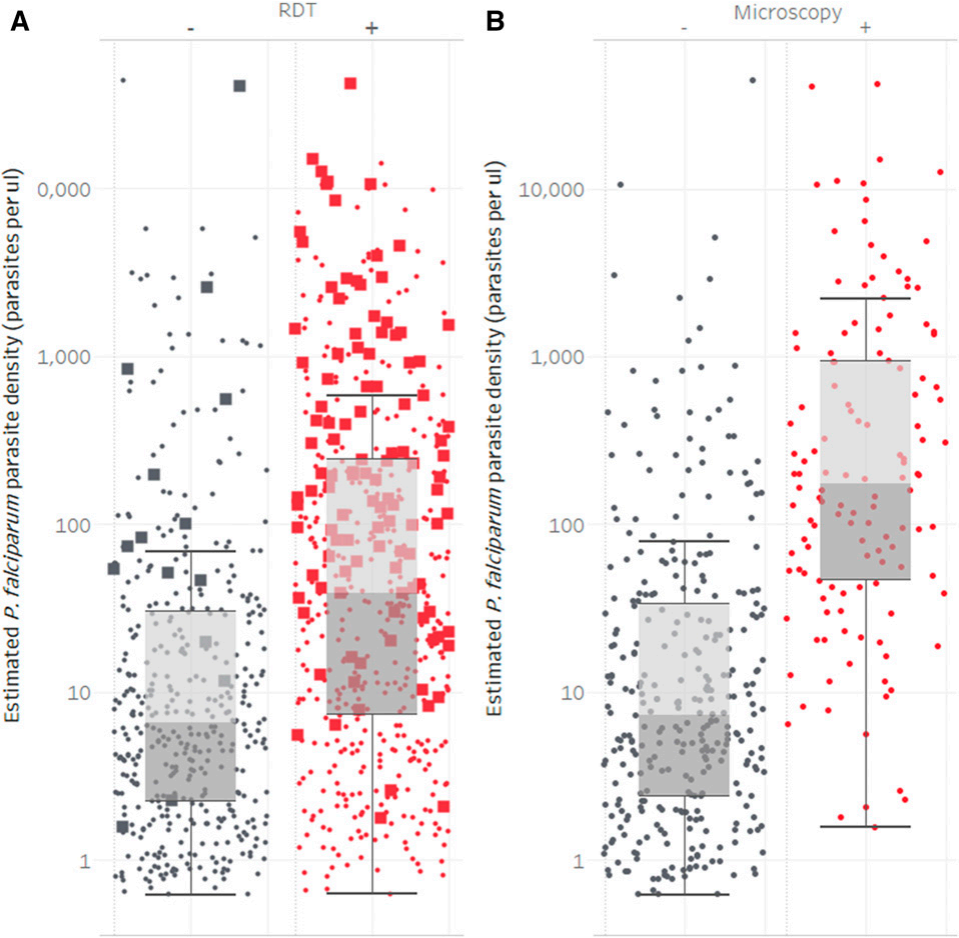
Parasite density of *Plasmodium falciparum* photo-induced electron transfer–PCR–positive samples stratified by the rapid diagnostic test result (**A**) or the microscopy result (**B**). Diagnostic results are shown as positive (red) or negative (gray). Microscopy positive samples (**A**) are shown as solid squares, while microscopy negative or samples not assessed by microscopy are shown as solid circles.

To assess whether the sensitivity of the two diagnostics was influenced by parasitemia of an infection, real-time PCR–generated crossing point values were converted into an estimated measure of parasite density and stratified by the RDT or microscopy result. For both diagnostics, there was a statistically significant difference in parasite density for samples testing positive by RDT (*P* < 0.001, Figure 1A, geometric mean of 47.7 (95% CI: 38.6–58.9) parasites per µL) or microscopy (*P* < 0.001, Figure 1B, geometric mean of 200 (95% CI: 137–293) parasites per µL) versus those testing negative (geometric mean of 10.3 (95% CI: 8.5–12.5) and 10.8 (95% CI: 8.7–13.5) parasites per µL, respectively).

To evaluate for any association between the test results by PCR and and those by RDT from 1 month to the next, all individuals with both RDT and PCR results for consecutive months were assessed (Table 3). For clarity, combined PCR and RDT results, where PCR is the gold standard, are referred to as true negative (TN) (i.e., RDT−/PCR−), false positive (FP) (i.e., RDT+/PCR−), false negative (FN) (i.e., RDT−/PCR+), or true positive (TP) (i.e., RDT+/PCR+). Where appropriate, the timing of samples is indicated in superscript as current month (^curr^) or previous month (^prev^).

**Table 3:** Comparison of previous months’ combined RDT and PCR results against the next months’ combined results for RDT and PCR

Previous month’s result	Next month’s result			
	True negative (%)	False negative (%)	False positive (%)	True positive (%)
True negative	22,981 (97)	212 (1)	195 (1)	185 (1)
False negative	228 (70)	51 (16)	14 (4)	31 (10)
False positive	242 (70)	8 (2)	70 (20)	28 (8)
True positive	245 (61)	28 (7)	58 (14)	72 (18)

RDT = rapid diagnostic test. All samples with both RDT and PCR results for at least two consecutive months are included. Figures in parentheses show the row percentage breakdowns of the previous month’s result combination by the next month’s result combination. True negative: RDT−/PCR−, false negative: RDT−/PCR+, false positive: RDT+/PCR−, and true positive: RDT+/PCR+.

**Figure 2.**
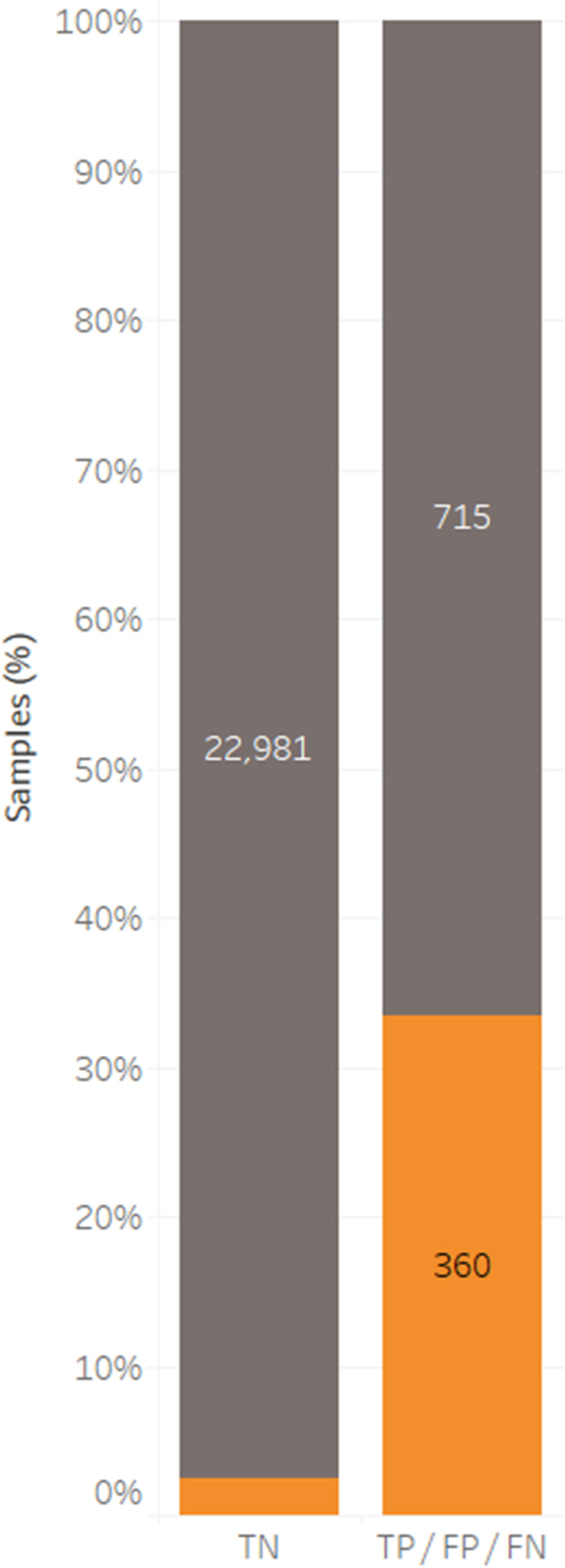
*Plasmodium falciparum* infections identified by both the rapid diagnostic test (RDT) and photo-induced electron transfer–PCR for all samples where both test results are available for two or more consecutive months. Combined results for the current month are shown as true negatives (gray) or positives by the RDT (false positive) or PCR (false negative) or PCR and RDT (true positive) (orange), stratified into the same groups for the previous month. Samples are expressed as a percentage of the column total, with inset figures showing the number of samples.

**Figure 3.**
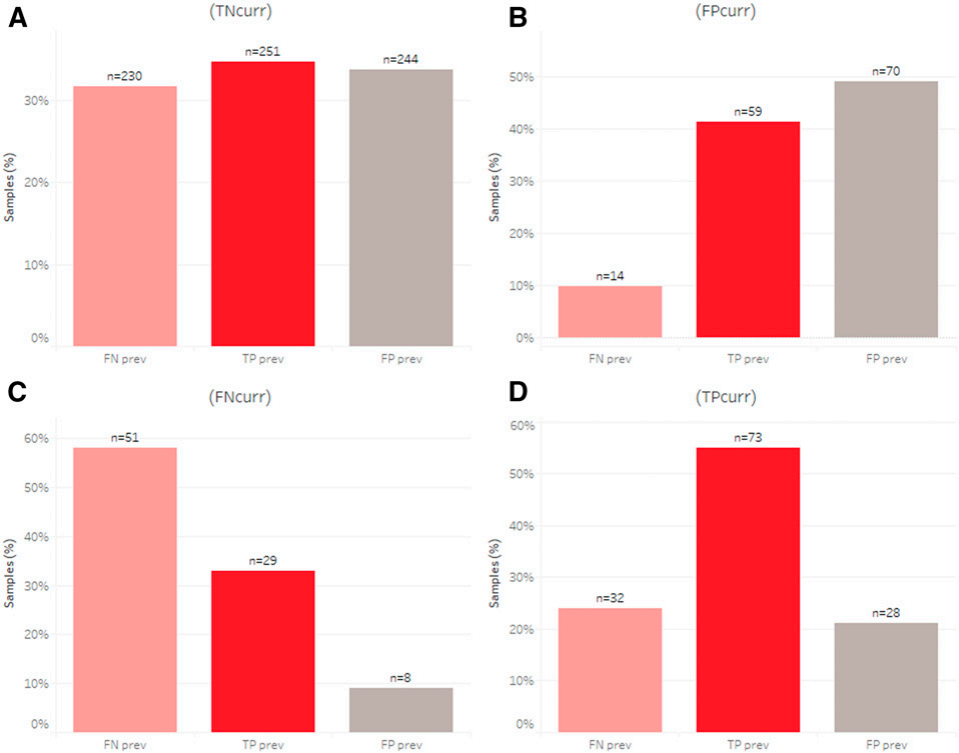
*Plasmodium falciparum* infections identified by both the rapid diagnostic test (RDT) and photo-induced electron transfer-PCR for all samples where both test results are available for two or more consecutive months. The breakdown for the current month, true negative^curr^ (**A**), false positive^curr^ (**B**), false negative^curr^ (**C**), or true positive^curr^ (**D**) is shown in each of the 4 panels. Bars are colored as false negatives (pink), false positives (gray), or true positives (red) for the previous month, and expressed as a percentage of the total (excluding true negative^prev)^, with inset figures showing the number of samples.

Among individuals who tested TN in the previous month (TN^prev^), roughly 3% tested positive by RDT and/or PCR in the next month. By comparison, among individuals who tested positive by the RDT and/or PCR in the previous month, 33.5% tested positive by the RDT and/or PCR in the next month (Figure 2). When examining the association between previous month’s status and the current month’s status, a few key observations were made (Figure 3). First, the odds ratios of being FP^curr^ are 20.2 (14.7–27.4), 30.1 (22.3–40.5), or 5.4 (3.0–9.1) times greater for those who were TP^prev^, FP^prev^, or FN^prev^, respectively, than those who were TN^prev^ (Figure 3B). Second, the odds ratios of being FN^curr^ are 8.2 (5.4–12.2), 2.6 (1.2–5.0), or 20.6 (14.7–28.4) times greater for those who were TP^prev^, FP^prev^, or FN^prev^, respectively, than those who were TN^prev^ (Figure 3C). Finally, the odds ratios of being TP^curr^ are 27.5 (20.4–36.7), 11.1 (7.2–16.4), or 13.4 (8.8–19.6) times greater for those who were TP^prev^, FP^prev^, or FN^prev^, respectively, than for those who were TN^prev^ (Figure 3D).

### All *Plasmodium* species.

After all samples were assayed by duplex PET-PCR for all *Plasmodium* spp. and *P. falciparum*, 87 DBS samples were found to be positive by PCR for the presence of a non–*P. falciparum* malaria parasite. Of these, eight of the samples could not be tested. For the remaining 79 samples, we were only able to positively identify a non–*P. falciparum* infection in 17 samples, meaning that 62 samples remained genus positive, but were not positive for any of the other species that we tested (*P. malariae*, *P. ovale*, and *P. vivax*). The PET-PCR crossing point values for the remaining samples were not significantly higher than those that were resolved. As with the non–*P. falciparum* infections, all *P. falciparum*–positive samples were tested and eight coinfections were identified (Table 4). No samples were found positive for *P. vivax*.

**Table 4:** Number and frequency of different mono- and coinfections with *Plasmodium* spp. determined by photo-induced electron transfer-polymerase chain reaction

*Plasmodium* species	*P. falciparum* (%)	*P. ovale* (%)	*P. malariae* (%)
*P. falciparum*	962 (97.5)	–	–
*P. ovale*	1 (0.1)	10 (1.0)	–
*P. malariae*	7 (0.7)	0 (0)	7 (0.7)

*P. falciparum = Plasmodium falciparum*; *P. malariae = Plasmodium malariae*; *P. ovale = Plasmodium ovale*. Note that percentages are given as a fraction of all infections.

**Figure 4.**
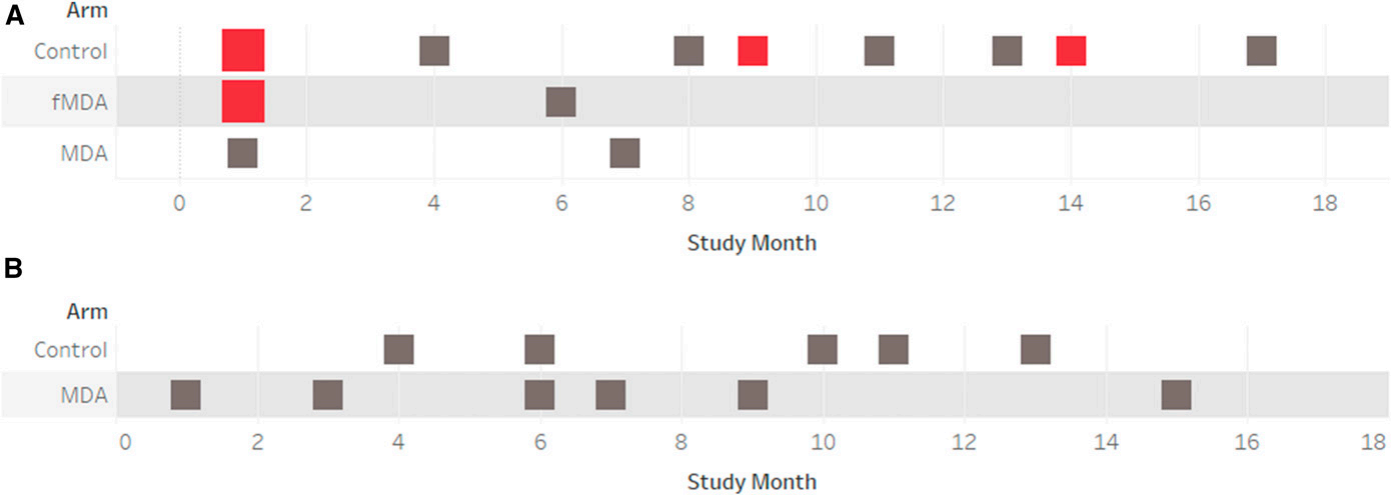
*Plasmodium malariae* (**A**) and *Plasmodium ovale* (**B**) infections identified by photo-induced electron transfer-polymerase chain reaction per month by trial arm in the cohort for the duration of the study. Infections are shown as rapid diagnostic test (RDT)–positive (red) or RDT-negative (gray), and the size of the square denotes the number of infections (small = 1, large = 2). No *P. ovale* infections were found in the focal mass drug administration arm of the trial.

For *P. malariae*, a total of 14 infections were identified with a peak of four infections in the first month, after which a maximum of one infection was identified per month (Figure 4A). Of the 14 individuals found with *P. malariae*, none were positive more than once. Fifty percent were coinfections with *P. falciparum* (Table 4), of which a number were RDT positive (Figure 4A). In the MDA and fMDA arms of the study, *P. malariae* infections essentially disappeared soon after the first two campaign rounds were completed, whereas in the control arm, *P. malariae* infections were found until the last month (Figure 4A).

By contrast, roughly 90% of all *P. ovale* infections were mono-infections (Table 4), none of which were RDT positive (Figure 4B). A total of 10 individuals were infected and, unlike *P. malariae*, one individual was found to be infected twice (11 infections total), with a gap of 1 month between the two positive results. *Plasmodium ovale* infections were only found in the MDA and control arms of the study, but the frequency of infections remained constant throughout the study (Figure 4B).

## DISCUSSION

Rapid diagnostic tests have revolutionized routine diagnostic confirmation of malaria as an illness. They are robust, inexpensive, easy to use, and very effective at identifying symptomatic infections. Increasingly though, in low-transmission settings, they are being used not only to diagnose clinical illness, but also, in other program activities such as reactive case detection, to find additional, often asymptomatic, individuals. These often low-parasitemia infections are often missed by standard RDTs, threatening elimination efforts.^13,14^ This study sought to both assess RDT performance and identify non-*falciparum* infections in an area that experienced a dramatic reduction in malaria prevalence over the course of an 18-month period.

Using PCR as the gold standard, overall there was very high (> 97%) concordance between the PCR and RDT results. Microscopy was only used in the first 6 months of the study but showed a similar pattern. High concordance was reflected in the excellent specificity and NPVs of approximately 98.5% for the RDT. However, the PPVs and sensitivity (approximately 53%) were lower than expected. Several factors may have contributed to both the false-positive and FN results.

**Figure 5.**
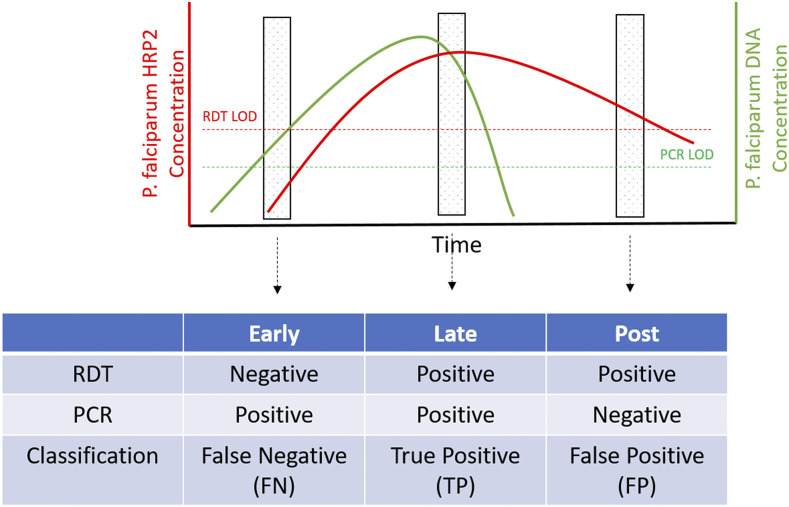
Schematic of assumed infection progression and associated diagnostic outcomes. Early in the infection, HRP2 concentration is below the RDT limit of detection (LOD), whereas *Plasmodium falciparum* DNA is above the PCR LOD, giving a false-negative result. Later in the infection, sufficient HRP2 has accumulated to yield a positive rapid diagnostic test (RDT), yielding a true positive. Finally, in the posttreatment/clearance phase, parasite DNA is absent, whereas HRP2 persists, giving a false-positive RDT result. Note that progression of an infection through the above stages is not linear or absolute, that is, a false-negative infection may never develop to a true positive.

To assist with visualizing the FN/TP/FP classification used in this study, a schematic (Figure 5) illustrates how a simple infection could potentially progress in terms of diagnostic outcomes. Clearly, each infection is a complex interplay between host, parasite, and diagnostic performance. As such, infections will progress or oscillate through these stages at very different rates.

### False-positive RDT results.

Rapid diagnostic tests assay for the presence of a parasite protein. The majority of *P. falciparum* RDTs measure the levels of *Plasmodium falciparum* histidine-rich protein 2 (*Pf*HRP-2), a protein that is well documented to persist many weeks after an infection has been cleared.^15^ To assess whether FPs were due to this persistent *Pf*HRP-2 antigenemia, previous month’s combined RDT and PCR results were assessed against the subsequent month’s results (Table 3, Figure 2). For individuals who were TP in the previous month, 14% were FP in the next (Table 3). This correlates to a 20.2 times greater odds ratio of being FP^curr^ for those who were TP^prev^ than those who were TN^prev^ (Figure 3B). Interestingly though, individuals who tested FP^prev^ had a 30.2 times greater odds ratio for testing FP^curr^ than those who were TN^prev^. Note that all RDT-positive individuals received a course of antimalarial treatment. This is markedly different from those who were TN (1%) or FN (4%) and suggests that persistent antigenemia is responsible at least in part for some of the FP RDT results. One would expect individuals who tested FP in the previous month to clear any *Pf*HRP2 by the next month. Interestingly though, 20% of those who tested FP in the previous month remained FP in the next. It is possible that this shows that *Pf*HRP2 is being cleared slower in this Zambian population and could be tested by measuring the antigen levels directly. Another possibility is that nonspecific cross-reactivity, as previously reported,^16^ may be contributing to this finding.

Although it would be optimal to reduce the number of FPs, these are relatively small numbers that are unlikely to translate to a significant increase in antimalarial consumption. Furthermore, they do not threaten the attainment of elimination, although they may briefly mask its arrival.

### False-negative RDT results.

Unlike FPs, FN RDT results represent a clear threat to interrupting transmission and if not minimized could prevent elimination from being reached as well as impact an individual’s access to prompt and appropriate treatment. It was therefore concerning to find that ∼45% (425/928) of PCR-positive individuals were RDT negative, resulting in a low RDT sensitivity (Table 1). However, this was tempered by ∼75% of these FN^prev^ individuals become aparasitemic (TN^curr^ or FP^curr^) in the following month (Table 3). In other words, most of these clinically undetectable infections appeared to resolve. Nevertheless, a significant 25% remained infected in the next month, of which two-thirds were undetectable by the RDT (FN^prev^). These individuals likely represent a chronically infected population that, despite their low parasitemias, could be contributing to ongoing transmission.

From a diagnostic perspective, it is likely that the key reason for the presence of FNs relates to the LOD of the RDT (i.e., samples have an *Pf*HRP-2 analyte concentration below the LOD. It is unlikely that the prozone effect, that is, the interference in a diagnostic sensitivity due to very high antibody or in this case antigen concentration, contributed significantly to the FN RDTs.^17^ Molecular techniques such as PCR have the potential to dramatically lower the LOD, and it is not surprising that they identify a significant number of FN RDT results. As with a previous study performed in Southern Province, Zambia, where RDT FNs were associated with a lower mean copy number,^18^ we also observed a significant reduction in parasite density in the FNs (Figure 1). This suggests that the FNs are explained at least in part by low-density infections. Clearly, if PET-PCR identifies FN RDTs, other even more sensitive nucleic acid–based amplification techniques with lower LODs will identify additional FN PET-PCR samples. A final consideration is that *Pf*HRP-2/3 gene deletions are contributing to the FN pool, and with deletions identified in a number of surrounding countries,^19–23^ it is highly likely that they are present in Zambia.^24^ In light of this, FNs represent an important set of samples to examine for any *Pf*HRP2/3 gene deletions.

To maximize test performance, one can either reduce the LOD (i.e., make the test more sensitive)^25^ or increase the volume of sample tested (i.e., increase the amount of the analyte sampled).^26^ For the former, this generally correlates with an increase in cost through additional assay steps, costlier reagents, supplementary oversight/controls, and high sensitivity to trace contaminants. In 2018, a new RDT with a significantly lower LOD was released to market. It is hoped that this next-generation test will improve detection rates, but it is not clear how well it will bridge the gap to the molecular approaches. For the latter, the volume of blood, usually 50–100 µL, that can be collected from a finger prick is limited, meaning that a significant proportion of the sample would be consumed in identifying a positive, thus leaving less sample for further analyses such as genotyping. Using a different blood collection system (e.g., intravenous) is time-consuming, costly, requires skilled personnel, and more onerous sample storage (e.g., cold chain). Moreover, this would be additional to the requirements of performing an RDT and may negatively influence consent for testing. For routine molecular surveillance, where emphasis has to be placed on a robust, rapid, and affordable (< $2 for commodities per sample by PET-PCR) system for sample collection and analysis, DBS combined with PET-PCR represents a good compromise to larger volume high-sensitivity approaches.

### Temporal heterogeneity.

Approximately 30% of individuals who tested positive the previous month, regardless of the test method, remained positive in the next month. When assessing TPs (TP^curr^, Figure 3D), an individual testing TP^prev^ is much more likely (odds ratio of 27.5) to test TP^curr^ than any other result from the ^prev^. Considering that all TP^prev^ individuals were treated with an appropriate course of antimalarial drugs, one would expect these to be the least likely to test TP^curr^. There are a number of potential explanations for this result. The first is that this represents a pool of individuals who are being repeatedly reinfected. Identifying and intervening in this high-risk population may be key to interrupting transmission. Hazard ratios and an adjusted negative binomial regression model identified PCR positivity at baseline as being significantly associated with reduced time to first infection and cumulative infection incidence, respectively (*P* < 0.01).^11^ The second is that there was a clinical treatment failure, which could be from suboptimal dosing (e.g., incorrect dosing or a failure by the patient to complete the full course) or from antimalarial drug resistance. To differentiate between a treatment failure and a reinfection, samples can be genotyped; however, although we did successfully genotype a number of infections, no consecutive TP samples were successfully genotyped and no clonal haplotypes identified. We therefore cannot rule out either option, but in light of the experience of health workers in prescribing antimalarials and encouraging adherence and the lack of documented drug resistance to the current frontline antimalarials in Zambia, we favor reinfection as the likely cause of this result.

### Other *Plasmodium* infections.

Although species identification has not been performed systematically across Zambia, *P. falciparum* dominates the landscape with ∼98% of all infections, *P. malariae* is consistently found in 2–4% of all infections, while *P. ovale* is rarely observed, and *P. vivax* essentially absent.^27–30^ This study broadly confirmed these observations, with *P. falciparum* representing 97.5% of all infections and small numbers of infections from *P. ovale* and *P. malariae* (Table 4). It is unclear at this stage why a large proportion of non–*P. falciparum* infections could not be resolved to the species level. To address this, we are exploring the utility of alternative PCR assays with a lower LOD, to both confirm the genus-positive result and to confirm the species if positive. With such a focus on *P. falciparum* detection in Zambia (e.g., the exclusive use of a *P. falciparum*–only RDT), it is encouraging that no samples were positive for *P. vivax* and only limited evidence of other non–*P. falciparum* infections was found. Nevertheless, from an elimination perspective, it is important to ensure that other species are also effectively targeted. Despite the small numbers, MDA and fMDA appeared to reduce the prevalence of *P. malariae* in comparison to the control arm (Figure 4A). Considering that 50% of *P. malariae* infections were coinfections with *P. falciparum* (Table 4), this species may be more amenable to *P. falciparum* elimination/control efforts, as identifying the *P. falciparum* reservoir will automatically identify half the *P. malariae* reservoir. By contrast, *P. ovale* was identified as a mono-infection 90% of the time (Table 4). While marginally rarer, *P. ovale* showed no obvious change in prevalence during the study. However, it was noticeably absent from the fMDA arm for unknown reasons. Cross-sectional surveys have continued in the trial areas and should be analyzed for non–*P. falciparum* species to increase confidence in confirming longer term trends.

## SUMMARY

Considering that the RDTs used in this study were developed to identify malaria in symptomatic individuals rather than to detect all infections, overall they performed well and demonstrated high specificity. However, sensitivity was significantly lower than expected and was associated with reduced parasite density, suggesting that this reflected the test’s LOD. A small pool of individuals were associated with multiple *P. falciparum* infections. Identifying these individuals and treating them for, or protecting them from, infection would be necessary for a more targeted approach. Finally, the low frequency of non–*P. falciparum* infections reflects current understanding of species transmission in Zambia, with *P. falciparum* dominating the landscape.
